# UNDERSTANDING FACILITATORS AND BARRIERS TO PARTICIPATION IN EVIDENCE-BASED FALL-PREVENTION INTERVENTIONS

**DOI:** 10.1093/geroni/igz038.2095

**Published:** 2019-11-08

**Authors:** Janice Mark, Gwen Bergen, Briana Moreland

**Affiliations:** 1 American Association of Colleges in Nursing, Washington DC, United States; 2 Centers for Disease Control and Prevention, Atlanta, Georgia, United States

## Abstract

Prescribing evidence-based interventions based on older adults’ modifiable risk factors is recommended to prevent falls. Older adults need to adhere to the prescribed intervention to successfully reduce risk. This study reports on a structured systematic review to understand patient attitudes to adherence to fall prevention interventions. A systematic search for publications from 2008-2018 identified 72 articles on patient attitudes toward exercise, physical therapy, medication management, podiatry, and vision care for fall prevention. Three reviewers coded facilitators and barriers based on a socio-ecological model of interpersonal, intrapersonal, community, and policy factors. Perceived susceptibility to falling and perceived effectiveness of the intervention were important factors across all fall prevention interventions. Physician prescribing and discussion facilitated exercise, medication changes, and physical therapy. For attitudes related to community and policy, the most reported barriers were transportation and cost. Information from this review can be used to improve the implementation of fall prevention interventions.

